# Old and modern antibiotic structures with potential for today’s infections

**DOI:** 10.5599/admet.1272

**Published:** 2022-03-04

**Authors:** David J. Newman

**Affiliations:** NIH Special Volunteer, Wayne, PA 19087 US. Email: djnewman664@verizon.net

**Keywords:** Peptides, Synthesis, Pathogens

## Abstract

Due to the lack of new antibiotics with efficacy against the ESKAPE and other resistant microbes, coupled to the demise of major pharmaceutical company antibiotic discovery programs, due to a number of factors but mainly ROI calculations and the lack of efficacy of combinatorial chemistry as a substitute, the search for novel antibiotics may well have moved to the utilization of older structures with significant synthetic chemistry input. This short review demonstrates how modern synthetic chemistry, when applied to either modification of current resistant antibiotics such as glycopeptides, or production of novel peptidic agents based on natural product sourced antimicrobial peptides (AMPs) and other potential initial peptide-based agents from genomic searches and baiting techniques, have produced active agents of significant utility. In addition, synthetic chemistry practitioners have now shown that they can produce bioactive molecules of greater than 800 Daltons in kilogram quantities under cGMP conditions.

## Introduction

With the advent of what might be called the “Dangerous Sextet” of current bacterial infections viz *Enterococcus faecium, Staphylococcus aureus, Klebsiella pneumoniae, Acinetobacter baumannii, Pseudomonas aeruginosa*, and *Enterobacter spp*. (aka the ESKAPE pathogens), came to the frequently belated realization that the antibiotics in current use were not effective as single agents against most, if not all, of these organisms. In a few advanced countries, expensive antibiotic drugs with suitable activities might be available, but these infections ignore economic boundaries. Thus, it behooves researchers to reinvestigate and/or modify agents that, in earlier days, were ignored due to their toxicities, difficulty in dosing, chemical stability or side effects due to their unknown interactions.

The World Health Organization included the organisms above in their listing of dangerous microbes, as shown below. On the 17^th^ of February 2017, they published a list of twelve microbes that were of concern as they were all resistant to a significant number of antibiotics then in current use. This list was meant to be a “wake-up call” to the pharmaceutical industry/scientists involved in the discovery of new antibiotics, be they natural products, semisynthetic or totally synthetic entities, by showing how widespread resistance is. On the list (www.who.int/mediacentre/news/releases/2017/bacteria-antibiotics-needed/en/),

- the first three (priority 1 listed as critical) were the Gram-negative organisms *Acinetobacter baumannii* (carbapenem-resistant); *Pseudomonas aeruginosa* (carbapenem-resistant); *Enterobacteriaceae* (carbapenem-resistant, ESBL-producing).
- The next 6 (priority 2 listed as high) contained two very well-known Gram-positive pathogens, *Enterococcus faecium* (resistant to vancomycin) and *Staphylococcus aureus* (MRSA), and four Gram-negative pathogens. *Helicobacter pylori* (clarithromycin-resistant); *Campylobacter spp*. (fluoroquinolone-resistant); *Salmonellae* (fluoroquinolone-resistant) and *Neisseria gonorrhoeae* (cephalosporin-resistant, fluoroquinolone-resistant).
- The last three (priority 3 listed as medium) were the Gram-positive *Streptococcus pneumoniae* (penicillin-non-susceptible) and the two Gram-negative *Haemophilus influenzae* ampicillin-resistant) and *Shigella* spp. (fluoroquinolone-resistant).

What will be described in this article are examples culled from the recent literature where elegant chemistry and modernized microbiological techniques have led to modifications of old agents and/or the administration of “adjuncts” that has enabled the renewed usage of agents that in the “halcyon days of yore”, which covered from the early 1960s until the effective elimination of large-scale antibiotic discovery programs in “Big Pharma”, which with a few exceptions would be pre-1990.

## The demise of combinatorial chemistry as a source of antiinfective lead structures

It would be worth commenting here that possibly the major “player” in the elimination of searching for microbial-sourced agents by “Big Pharma” was the concept of large-scale to very large scale (>10^6^) syntheses of small compounds via combinatorial chemistry. The idea was that these agents would be patentable and “easily synthesized”, rather than have the large-scale systems necessary for natural product sourced agents. In addition, a major problem with microbial sourcing (the major source used in all earlier cases) was the task(s) associated with what became known as “dereplication”, or the ability to rapidly decide whether an isolated compound was new or old and often known under a variety of names. It should be noted that the advent of >100 MHz NMR, portable computers, accessible compound databases and automated HPLCs were way in the future, even in the early 1980s. The irony in the early days of combichem was that the original “successes quoted for increased bioactivity” used peptide-based molecules or small compounds based on bioactive natural products as their starting points.

An excellent example of what happened when combichem structures were substituted for products from natural sources and/or their derivatives was the review in 2007, giving the total lack of success when massive numbers of combichem structures met up with ~70 isolated targets at GSK [1]. I should point out at this stage that I was part of the earlier SK&F antibiotics program based on microbial sources and modification of beta-lactam based molecules from 1976 to its sudden demise in April of 1985. That program was certainly more successful than its subsequent descendants. A 2015 paper by Blaskovich et al. gives more of the subsequent history of the demise of promising antibiotic programs by large pharma in the subsequent years to 2015 [2].

In the following sections, I will illustrate how, by building upon “old or at times new structures” by competent scientists (chemists and microbiologists), and/or by utilizing modern techniques based on earlier systems, old structures suddenly became significant candidates for clinical trials, and nowadays utilization of the enormous capabilities of genetic manipulation can lead to novel agents with the desired attributes against the ESKAPE pathogens and their subsequent descendants.

## Vancomycin and other glycopeptides; Initial and later history

For a significant number of years, vancomycin (**1**) and then later its close chemical relatives were often defined as the “antibiotic of last resort”. Vancomycin was originally introduced into clinical medicine by Lilly in the middle to late 1950s, though there is a debate in the literature as to the actual date. The Integrity^R^ database (now Clarivate Analytics) uses 1955, but Butler et al. in a review in 2014 [3] used 1958. as the FDA approval date. However, the actual structure was not fully defined until 1982 when the presence of asparagine was confirmed [4].

Resistance to vancomycin occurred relatively early, but before the advent of advanced structural techniques, including amino acid analyses of Gram-positive cell walls, the reason for the resistance was not known. With the advancement of knowledge, it became apparent that vancomycin (and its later naturally occurring or semisynthetic “chemical cousins”) inhibited bacterial growth by binding to the *L*-Lys-*D*-Ala-*D*-Ala-CO_2_H terminals in the cross-links in the Gram-positive cell wall. Subsequent work demonstrated that the *Van^R^* phenotype was as a result of the simple change to *D*-Lactate in place of the terminal *D*-Ala residue in the cases of *vanA*, *vanB, vanD* and to *D*-Ser for *vanC, vanE, vanG* clinical phenotypes. In addition to vancomycin resistance, *S. aureus* cultures that were classified as Methicillin Resistant (MRSA), were also becoming vancomycin-resistant as well. The vancomycin resistance level due to the *D*-Lac substitute was approximately 1000-fold and for the *D*-Ser modification approximately 140-fold.

Interestingly, the idea that resistance was due to the use of glycopeptide antibiotics in animal feeds that then led to the resistance seen was shown to be inaccurate by two reports. One in 2011 by D’Costa et al., [5] and the other by Wright et al. [6] the following year, both papers demonstrating that microbes over 10,000 years old isolated from Yukon ice fields had similar resistance phenotypes.

In the last few years, three semisynthetic glycopeptides entered clinical use in the USA and other countries. In 2009 telavancin (a close relative of vancomycin) was approved by the FDA and then in 2014, dalbavancin, which was derived from part of the A40926 complex, and oritavancin derived from chloroeremomycin were approved. The drug complex teicoplanin, which is a mixture of closely related compounds, is used in Europe and interestingly, there are some *VanR* phenotypes that are not resistant to this mixture and the reverse also occurs, though in general, most strains are resistant to all.

### Synthetic modifications of vancomycin

The laboratory that has had the greatest effect on “modifying” the basic structure of vancomycin (**1)**, with the aims of reducing resistance and potentially expanding the range of microbes that it can “inhibit”, is, without doubt, the Boger laboratory at the Scripps Research Institute in La Jolla, California. Over the last ten plus years, that laboratory has published papers showing how by making internal modifications to the peptide backbone in this molecule, that were simple in concept but required very clever synthetic chemical processes to achieve, have led to a series of related vancomycin molecules that demonstrated very significant antibiotic activities against MRSA and *E. faecalis* (both *VanA* & *VanB* phenotypes) depending upon very simple changes in one position. Then the Boger group extended the synthetic chemistry to other parts of the base molecule by adding small parts from other glycopeptides in clinical use.

Structure (**2**), which was redrawn from the Proceedings of the National Academy of Science paper by Okano et al. [7] shows the substitutions used. The compilation of vancomycin structural changes and the tables in that report demonstrated how these various modifications “converted” total resistance to *E. faecalis* and *E. faecium* (MICs of vancomycin > 250 μg mL^-1^) to molecules with MICs from 5 to 0.005 μg mL^-1^ for these *Van* A/E resistant microbes.

Then in 2020, the same group reported the results of further guanidino modifications on the C-terminus of vancomycin that improved antimicrobial activity and appeared to provide a synergistic mechanism of action independent of *D*-Ala-*D*-Ala [8]. By using structure (**2**) as the base, then coupling the 4-chlorobiphenyl)-methyl (CBP) modification, which they had previously shown to give significant increases in activity, with X = O and R = a variety of guanidino substituents, they produced a series of relatively simple modified vancomycins that displayed sub-microgram activities (MIC levels) against significant vancomycin-resistant clinical specimens.

The following is a direct quote from that paper. “a prototypical member of the series, G3-CBP-vancomycin (15) exhibits no hemolytic activity, displays no mammalian cell growth inhibition, possesses improved and especially attractive *in vivo* pharmacokinetic (PK) properties, and displays excellent in vivo efficacy and potency against an especially challenging multidrug-resistant (MRSA) and VanA vancomycin-resistant (VRSA) *Staphylococcus aureus* bacterial strain.”

**Figure 1.**
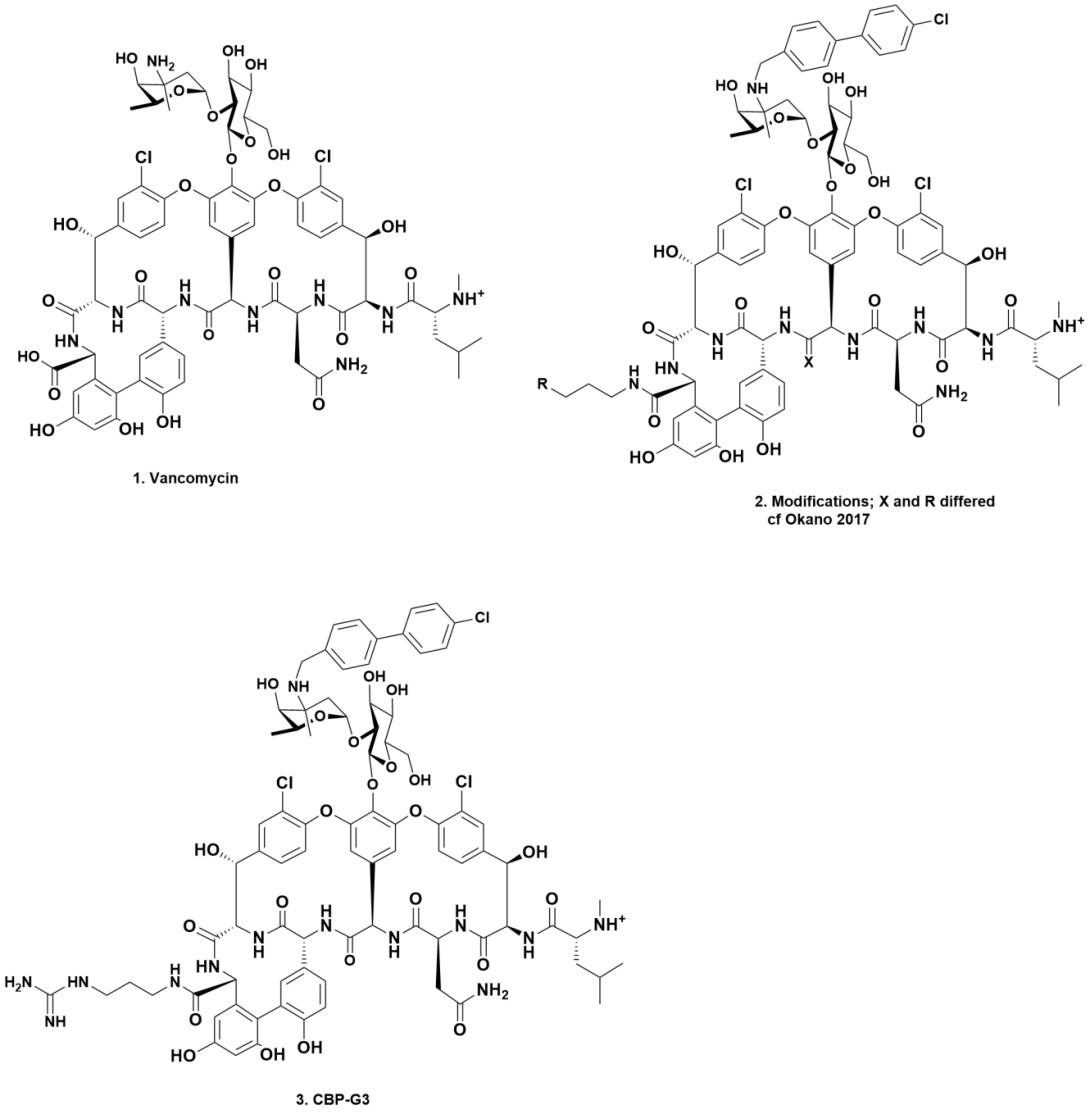
Vancomycin and Boger Lab Modifications; Structures **1** to **3**

The structure (15) mentioned in the direct quote above from Wu et al. [8] is shown as structure (**3**) in Figure 1.

In addition to the papers referred to above, another recent paper from the Boger lab [9] gives an excellent precis of the modified vancomycin derivatives mentioned above, plus others from the Boger lab. They comment that these agents are now known in that laboratory as “maxamycins”. This paper is well worth reading to gain further insight into these chemical modifications of a microbial product that first saw “light of day” in the middle to late 1950s, in order to overcome microbial resistance.

## A comment on modern synthetic methods as a route(s) to cGMP product(s)

**Figure 2.**
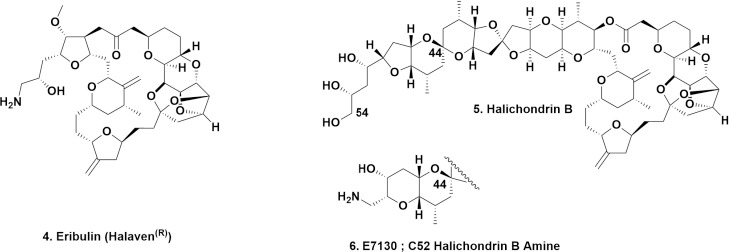
Halichondrin B and Synthetic Derivatives; Structures **4** to **6**

It should be emphasized at this juncture that synthetic organic chemists have succeeded in the last few years in producing large quantities of cGMP-quality, partial or complete natural products that have either become drugs, eribulin (**4**) MW 730 being the prime example utilizing data from the Kishi synthesis of halichondrin B (**5**) MW 1111. Or nowadays as potential leads to drugs, with the total synthesis of a derivative of halichondrin B E7130 (**6**); MW 1111 at the 10-gram level, reported by the same Eisai group that synthesized eribulin [10], which is currently in Phase I clinical trials. These structures are shown in Figure 2.

### Tetracycline-like molecules

Rather than cover the modified tetracyclines Omadacycline, Eravacycline and Saracycline (structures not given), that came from the Lederle (then Wyeth and now Pfizer) laboratories in the 1990s with these variations being approved as drugs in 2018, there were two compounds reported that had the basic nucleus of the tetracyclines but with extensions on the base four-ring scaffold. These compounds were the Viridicatumtoxins, now known as A (**7**) and B (**8**). They were first reported by Hutchinson et al. in 1973 from the fungus *Penicillium viridicatum* [11] as Viridicatumtoxin. Subsequently the B variant was reported by Zheng et al. in 2008 [12], and the synthesis of the B (**8**) variant was originally reported by the Nicolaou group in 2013, [13] with a subsequent structure revision and syntheses of related “microbiologically active compounds” in 2014 [14]. This molecule (as the mixture of enantiomers) was significantly active against resistant strains of *E. faecalis, E. faecium* and MRSA, and the natural A isomer was comparably active against the same strains.

Recently the biosynthtetic gene cluster (BGC) for Viridicatumtoxin was identified in *A. nidulans*, a fungus not previously reported to produce this agent. That report also identified the same cluster in *P. brasilianum* and in three other *Aspergillus* spp [15]. Interestingly, the Capon group at the University of Queensland reported five years earlier in 2015, that the *Paecilomyces* sp., known as CMB-MF010, which they had isolated from the inner tissues of the intertidal pulmonated mollusk *Siphonaria* sp., yielded viridicatumtoxins A (**7**) and B (**8**) and the new viridicatumtoxins C- F (**9-12**), and spirohexaline (structure not given). Of these compounds, viridicatumtoxins B (**8**) and C (**9**) demonstrated the largest separation of antimicrobial activity versus cell-line cytotoxicity with between 15-to-40-fold differences in favor of the antimicrobial activity. Of the six Viridicatumtoxins, A (**7**) and B (**8**) were the most active against MRSA and vancomycin-resistant *E. faecalis*. What is also of interest is that the Capon group demonstrated that “regular tetracyclines” could be transformed by ring-opening by the *Paecilomyces* sp., mentioned above, perhaps demonstrating a resistance methodology by fungi against these molecules, since none of the modified agents demonstrated any significant bioactivity [16].

In 2020, the antibacterial mechanism of these agents was proven by Li et al. in a report in ACS Infectious Diseases [17]. This report confirmed that, as reported earlier, this series of molecules bind directly to the undecaprenyl pyrophosphate synthases (UPPS) of *E. faecalis, S. aureus* and *E. coli* in a direct and high-affinity manner. This paper is worth consulting as the models can be used for further optimization of these molecules and analogues as UPPS is an essential component of cell wall biosynthesis.

**Figure 3.**
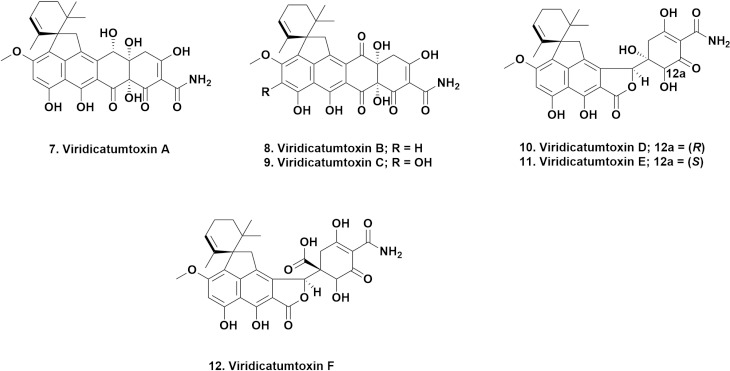
Viridicatumtoxins; Structures **7** to **12**

Since the Nicolaou group have successfully synthesized these molecules, viridicatumtoxin A (**7**) and B (**8**) and the Capon group have shown the simple difference in C (**9**), these are excellent candidates for large scale production, synthetically or genomically via modification of the BGC plus fermentation, to provide another candidate for use against members of the Gram-positive ESKAPE series. The relevant structures are shown in Figure 3.

### Peptidic candidates (other than vancomycin)

This section will include both naturally occurring and partially synthesized molecules. In some cases, the molecules will be well-known and not in others.

### Colistins and polymyxins

These peptidic antibiotics were frequently the “bane” of microbiologists and chemists in the 1960s to late 1970s when pharmaceutical companies with active antibiotic search programs based on microbial fermentation were in vogue. This was because these compounds and their close chemical relatives, had “for then” significant toxicities when compared to the tetracyclines, aminoglycosides etc. They were usually just “tossed aside” and no further work was done with them, nor with the microbe(s) producing them.

With the current advent of multiple resistant microbes, combination therapy including these agents has been utilized, using both polymyxins and colistin (*aka* polymyxin E). However, in 2016, a report was published [18] giving details of a transmissible plasmid expressing the *mcr* gene. This gene encoded the phosphoethanolamine transferases (pEtN) that catalyzed the addition of a phosphoethanolamine moiety to lipid A in the outer membrane of Gram-negative bacteria, thus generating resistance to the antibiotics in current use, including these peptidic agents. A major problem with the bacteria that contain this plasmid (or close variants) is that they are easily transmissible as “water-borne” microbes, and thus have spread from their original source in mainland China, to become significant problems in multiple areas, as shown by the 2021 paper by Cherak et al [19].

In an effort to produce new variations on the old and well used/known polymyxin B and colistin, a group of researchers from China, South Australia and Germany recently (2021) published the results of a large-scale program to discover potential agents against resistant *A. baumannii* [20]. At the time of writing of that paper over 2000 polymyxin analogues had been reported in the literature, with some early and some more recent examples also reported by Vaara in 2019 [21]. Of the 2000 plus variations referred to earlier, only 3 demonstrated improved activities against Gram-negative pathogens.

Using clever chemistry and sequential alanine substitution, Jiang et al [20] derived a potent and novel polymyxin analogue, FADDI-287 (**13**) that folded similarly to PMB_3_ (**14**) and demonstrated MICs of 0.125-0.5 μg mL^-1^ against *A. baumannii* compared to polymyxin B. FADDI-287 is covered by the patent WO2015149131 which has also been granted in the USA and China.

Very recently, the Brady group presented details on a semisynthetic, optimized AMP based upon a genomic analysis of sequences in bacteria that might well produce agents that are analogues of colistin (**15**). They reported the modified colistin known as biphenyl macolacin (**16**) in Nature in early 2022 [22]. Interestingly a major substitution was the biphenyl group, one that Boger used in his successful synthesis of the highly bioactive maxamycins.

### Vancomyxins

Although this discussion could fit under the discussion of vancomycin modifications above, because it now extends the activity of vancomycin analogues into Gram-negative space, and does not come from the Boger group, it is probably a better fit in this section.

Although vancomycin by itself (as described earlier) is effectively inactive against Gram-negative bacteria, it will bind to the lipid II component in Gram-negative bacteria if the outer membrane is “made permeable.” It has been known since 1989 that coadministration of suitable “penetrating” agents with vancomycin will synergize the effects of vancomycin against the Gram-negative bacterium *E. coli* [23]. Then in 2010, Gordon et al demonstrated significant activity of a vancomycin-colistin physical combination (*i.e.*co-administration of the two antibiotics) [24].

Moving to 2021, van Groesen et al came up with what they are calling “Vancomyxins,” where a short spacer was inserted between vancomycin and what they called the “polymyxin E nonapeptide PMEN (**17**).” Depending upon the specific combination of these molecules, they could be linked via the C-terminus of vancomycin, or in other examples, linked to the vancosamine amino group via a slightly more complex method involving a triazole linker, giving rise to a series of compounds [25].

Their structures are not given due to their complexity/sizes, but the combinations were evaluated against four Gram-negative and five Gram-positive bacteria with vancomycin and PMEN separately, and then vancomycin plus a constant 8 μg mL^-1^ of PMEN versus six of the vancomyxins. In most cases, one or more of the vancomyxins demonstrated MICs of between 8 and 16 μg mL^-1^, against microbes that were resistant to the unpaired molecules with no activities at or below 128 μg mL^-1^ for the Gram-negative microbes except for one outlier, the *P. aeruginosa* strain (ATCC 27853) that gave the only positive result when the unlinked combination was used. As would be expected, against the Gram-positive microbes significant activities were seen, even as low as less than 0.008 μg mL^-1^ for an *S. simulans* strain that had an MIC of 0.125 μg mL^-1^ against vancomycin alone.

Thus, in contrast to the very interesting Boger compounds referred to earlier, simple combined “old” antibiotics did provide some excellent results. Their hemolytic and nephrotoxic activities were assessed by means of a viability assay using conditionally immortalized proximal tubule epithelial cells (ciPTECs), with relative mitochondrial activity after 24 h as the end point. In those assays, toxicity arose at concentrations well above the MIC values reported. However, *in vivo* activities and toxicities were not measured and would have to be performed in the future.

## Antibacterial-active cyclic peptides (excluding polymyxins and colistins)

What is not often realized is that the first natural product antibiotic to go into clinical use but not under terms equivalent to formal approval as a drug entity in the modern sense, was not penicillin as most people think, but the cyclic peptide tyrocidine and the probable second, a derivative of “gramicidin” that was initially mentioned in a 1940 report [26], with the structure of tyrocidine A (**18**), reported later in 1952 [27]. These two molecules are usually not mentioned in any discussion of early antibiotics. However, contrary to what most people in the West may think, the aminoglycoside streptomycin (**19**) was not the next antibiotic to go into use for battlefield wounds at around the same time as penicillin in World War II. The next antibiotic to go into use for these conditions was gramicidin S (**20**), first used by the USSR for battlefield injuries in 1943 [28,29]. Further reports in the same basic time frame from the then USSR, covered more clinical aspects [30,31]. Then in 1946, Gause discussed the large-scale production and chemistry of this agent [32]. Even today, variations on this cyclic peptide are still being investigated.

Very recently there have been three reviews relating to antimicrobial peptides from naturally occurring and synthetic sources. In 2020, an excellent review by Lazzaro et al [33] discussed the evolution of antimicrobial peptides. In this paper they suggested that there was evidence showing adaptive maintenance of polymorphism in AMP genes, and perhaps adaptative loss of AMP activity. They also suggested as shown earlier with the polymyxins/colistins, that synergy might well occur with “conventional antibiotics”. What is interesting, is that they show synergy between magainin 2 and the AMP PGLa [34] both of which are AMPs found in the skin of the African frog *Xenopus laevis* and eukaryotes can rapidly deploy multiple distinct classes of AMPs simultaneously. Although it was always thought that AMPS have broad spectrum activities, genetic disruption of AMP genes result in different responses to infections by *S. aureus* in the beetle *Tenbrio molitor*. Similar separation of activities/genes in other insects have caused an alteration in the idea that “broad spectrum responses” may not be the case every time, but specificity in responses appears to be one response across many eukaryotic phyla.

In 2021 a group from Malaysia [35] reported the results of a literature review from 2011 to 2021 using a variety of available databases. Table 1 in that review (which is an Open Access paper) covers analogies between naturally occurring and synthetic agents, with the rest of the review covering methodologies and some history of both natural and synthetic AMPs, with an up-to-date series of references [35].

Closely following the Malaysian paper was one from an Italian group covering semisynthetic and synthetic AMPs, with an extension into some with antifungal and antiviral activities [36]. This review finishes with a comment on the semisynthetic AMP Murepavadin (**21**), a cyclic beta hairpin peptidomimetic based on the antimicrobial peptide, protegrin (PG-1). It is nonlytic and targeted the LptD protein transporter in *P. aeruginosa*. It reached Phase III clinical trials as an IV-preparation against pneumonia, but in July 2019, the two trials were terminated due to high levels of unexpected kidney injury. In December 2020, the UK Medicines Agency permitted a Phase I trial to begin targeting oral inhalation route in people with cystic fibrosis who are infected with *P. aeruginosa*.

An excellent and very recent paper that covers some of the above examples, and other potential agents that can be targeted against the outer membrane(s) of Gram-negative microbes, is the review by Klobucar and Brown in 2022 that should be consulted as they cover other agents as well [37].

## Other peptide-based antibiotic classes with potential

Due to the ability today to synthesize natural-product derived agents, particularly peptidic molecules under GLP and when necessary, cGMP conditions in bulk, once a suitable compound has been identified by using up to date versions of the classical searching methods from microbes, they can be synthesized and/or modified as needed in order to extend testing schedules.

Two examples and three recent reviews will be discussed under this rubric. The first will be the material found by the Lewis’ group at Northeastern University, and the second will be what I call the “Grasslands DNA Program” at Rockefeller University.

### Teixobactin

In 2015, the Lewis group at Northeastern University reported the isolation of a previously unknown microbe that they discovered by use of what can be best described as a “baiting technique”, though it was similar in concept to a technique used years earlier by a company named “One-Drop” that was eventually purchased by Diversa, though details were lost when Diversa went under financially.

The compound that was isolated was active against multiply resistant Gram-positive bacteria, and it was found to target pyrophosphate and sugar moieties present in Lipid II and III. Lipid II is linked to peptidoglycan (target of the glycopeptide antibiotics) and the lipid III binding is linked to down-regulation of cell wall teichoic acid biosynthesis. Over the last few years, there have been three reports on the total synthesis of this agent, plus several dozen reports covering analogues and SAR studies as shown by references in Gao et al. [38], all using solid-phase techniques. In 2019, Gao et al. published the first solution-phase synthesis, thus adding to the synthetic repertoire for this agent and the potential for many analogues [38]. Thus there are at least two different routes to the compound and derivatives. An example of modifying the agent and adding activity against *P. aeruginosa* can be seen in the report by Ng et al. in 2018 [39].

### Grasslands DNA

In 2014, the Brady group at Rockefeller published an article in Current Opinion in Microbiology describing the varied then current methods that used metagenomic studies to obtain potential antibiotic information without isolating the microbe(s) [40]. In 2015 Charlop-Powers and Brady published an informatic package that linked geographic analysis and related microbiome data [41]. Following on, in 2016, the group published their initial data on “Urban Park Microbiomes” as sources of biosynthetic diversity (hence the title for this section) [42]. In this work, they reported a gene cluster that could be linked to a novel calcium-dependent antibiotic (CDA).

In 2018 they reported on the culture-independent synthetic-bioinformatic natural product (syn-BNP) discovery approach covering the survey of analogues of paenimucillin A (**23**) that led to the discovery of paenimucillin C (**24**) which inhibited the growth of multidrug-resistant *A. baumannii* isolates, including a rat cutaneous wound model, with mechanistic studies implying a membrane-associated mode of action [43].

In 2020 this report was expanded by the same group giving details of the synthesis of 157 cyclic peptides from 96 NRPS clusters. These compounds were then tested against the ESKAPE pathogens yielding nine antibiotics with activities against some of these microbes as well as *M. tuberculosis* [44].

That same year, the group reported on the culture-independent discovery of the CDA compound referred to in their earlier general paper in 2016, demonstrating excellent activities (MIC values between 0.1 to 0.8 μg mL^-1^) for malacidin A (**25**) depending upon the particular resistant organism tested. These included microbes resistant to β-lactams, tetracyclines, vancomycin and aminoglycosides. The other CDA that they reported, malacidin B (**26**), differs by one methylene group from malacidin A [45]. This paper has over 180 citations in Scopus as of the middle of January 2022. Then in 2020, the complete synthesis of malacidin A was published by a joint group from the University of Hong Kong and Rockefeller [46]. As a result of the malacidin findings, a second total synthesis of malacidin A and analogues was recently published by the Brimble group in New Zealand [47].

## Three recent reviews on other peptidic antibiotics

In 2021, two interesting reviews on peptidic antibiotics were published, with a third in 2022. The first in 2021 discussed emerging peptides with therapeutic potential, with one author from the biotech company Polyphor in Switzerland, which has been involved in clinical trials with such agents, demonstrating the variation in linear and cyclic peptides with significant bioactivities against resistant microbes [48]. The second in 2021, was from a group in China and an interesting part of that review is a long table listing natural, semisynthetic and synthetic agents reported in the last five years. That table makes interesting reading, as it aptly demonstrates what can be done with current synthetic methods [49]. The third, accepted at the very end of December 2021 and currently online, is full of up to date listings of agents, sources, and clinical trial results, plus discussions as to probable and actual mechanisms of action [50].

## Conclusion

Synthetic chemists have now shown that they can modify natural product-based antibiotics that have ceased to be viable due to the build-up of multiple resistance profiles. As an example of what can be done today by competent synthetic chemists, the recent review by Nicolaou and Rigel shows some of the capabilities of experts in this field [51]. To this review can be added the work referred to earlier on the total synthesis of a derivative of halichondrin B E7130 (MW 1111, **6**) producing 10 grams of a cGMP product [10], together with the production of 1 gram of cGMP Bryostatin 1 by the Wender group [52].

The compounds referred to in the previous paragraph and those alluded to in the body of the text are just some examples of the many totally synthetic methods described in the literature for bioactive agents from natural product sources in the relatively recent past, covering compounds in many biological areas. It should also be noted that semi-syntheses starting from a natural product precursor have been a routine technique practiced by many chemists, particularly with marine-sourced bioactive agents and have been covered in detail by many authors.

As a result, for “antibiotic discoverers/developers” of today, a close relationship between microbiologists, natural products and synthetic chemists may well be the current optimal linkage to find and subsequently develop novel agents with clinical relevance. In addition, the advent of searchable databases that cover compounds, activities and genomic information should also be part of the mixture required to discover and develop agents against the ever-increasing number of resistant microbes.

## Figures and Tables

**Figure 4.**
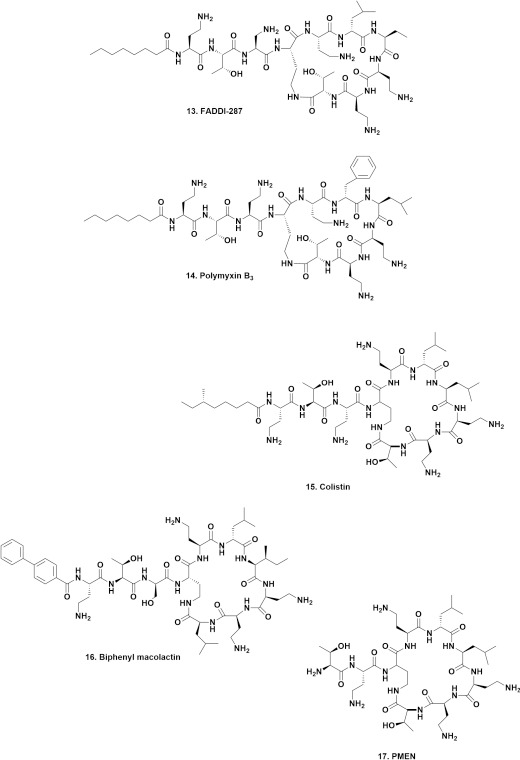
Colistin and Polymyxin Derivatives; Structures **13** to **17**

**Figure 5.**
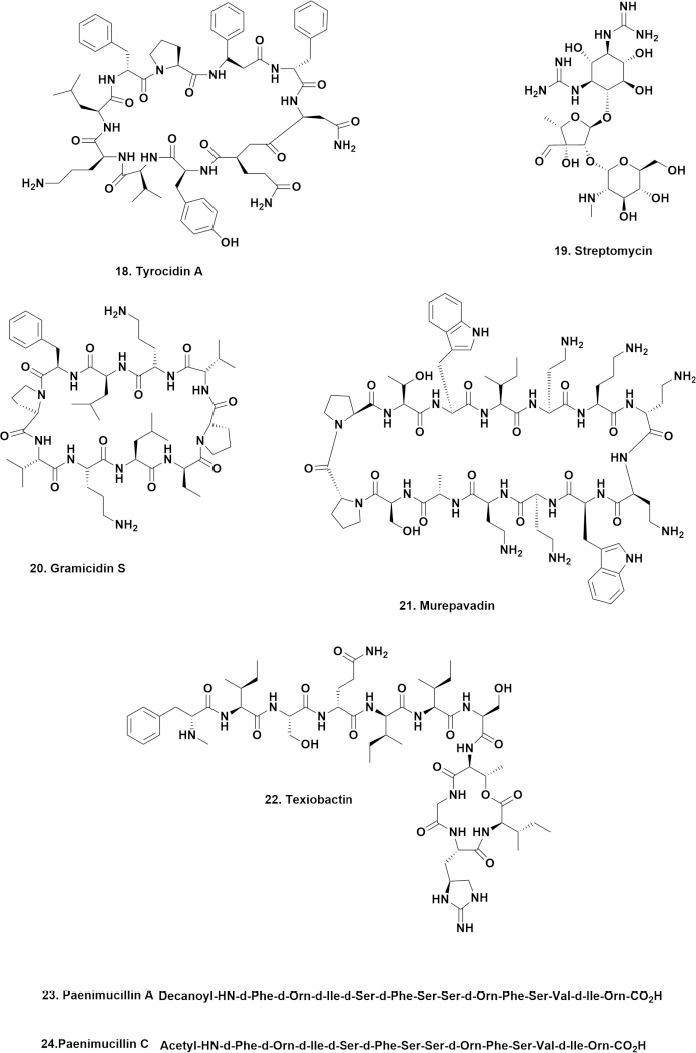
Early (1943) and Late (2010 Plus) Antibiotics; Structures **18** to **24**

**Figure 6.**
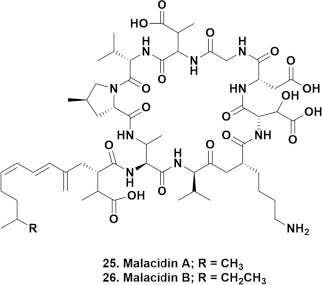
Malacidin A and B; Structures **25** to **26**

## References

[1] Payne D.J. Gwynn M.N. Holmes D.J. Pompliano D.L.. Drugs for bad bugs: Confronting the challenges of antibacterial discovery. Nat. Rev. Drug Discov. 6 (2007) 29-40. https://doi.org/10.1038/nrd2201. 10.1038/nrd2201 17159923

[2] Blaskovich M.A.T. Zuegg J. Elliott A.G. Cooper M.A.. Helping chemists discover new antibiotics. ACS Infect. Dis. 1 (2015) 285-287. https://doi.org/10.1021/acsinfecdis.5b00044. 10.1021/acsinfecdis.5b00044 27622818

[3] Butler M.S. Hansford K.A. Blaskovich M.A.T. Halai R. Cooper M.A.. Glycopeptide antibiotics: Back to the future. J. Antibiot. 67 (2014) 631-644. https://doi.org/10.1038/ja.2014.111. 10.1038/ja.2014.111 25118105

[4] Harris C.M. Harris T.M.. Structure of the glycopeptide antibiotic vancomycin. Evidence for an asparagine residue in the peptide. J. Am. Chem. Soc. 104 (1982) 4293-4295. https://doi.org/10.1021/ja00379a062. 10.1021/ja00379a062

[5] D'Costa V.M. King C.E. Kalan L. Morar M. Sung W.W.L. Schwarz C. Froese D. Zazula G. Calmels F. Debruyne R. Goldiing G.B. Poinar H.N. Wright G.D.. Antibiotic resistance is ancient. Nature Biotechnol. 477 (2011) 457-461. https://doi.org/10.1038/nature10388. 10.1038/nature10388 21881561

[6] Wright G.D. Poinar H.. Antibiotic resistance is ancient: implications for drug discovery. Trends Microbiol. 20 (2012) 157-159. https://doi.org/10.1016/j.tim.2012.01.002. 10.1016/j.tim.2012.01.002 22284896

[7] Okano A. Isley N.A. Boger D.L.. Peripheral modifications of [Ψ[CH2NH]Tpg4]vancomycin with added synergistic mechanisms of action provide durable and potent antibiotics. Proc. Natl. Acad. Sci., U.S.A. (2017) E5052-E5061. https://doi.org/10.1073/pnas.1704125114. 10.1073/pnas.1704125114 28559345 PMC5495262

[8] Wu Z.-C. Cameron M.D. Boger D.L.. Vancomycin c-terminus guanidine modifications and further insights into an added mechanism of action imparted by a peripheral structural modification. ACS Infect. Dis. 6 (2020) 2169-2180. https://doi.org/10.1021/acsinfecdis.0c00258. 10.1021/acsinfecdis.0c00258 32598127 PMC7429263

[9] Wu Z.-C. Boger D.L.. Maxamycins: durable antibiotics derived by rational redesign of vancomycin. Acc. Chem. Res. 53 (2020) 2587-2599. https://doi.org/10.1021/acs.accounts.0c00569. 10.1021/acs.accounts.0c00569 33138354 PMC7674238

[10] Kawano S. Ito K. Yahata K. Kira K. Abe T. A. T. Asano M. Iso K. Sato Y. Matsuura F. Ohashi I. Matsumoto Y. Isomura M. Sasaki T. F. T. Miyashita Y. Kaburagi Y. Yokoi A. Asano O. Owa T. Kishi Y.. A landmark in drug discovery based on complex natural product synthesis. Sci. Rep. 9 (2019) 8656. https://doi.org/10.1038/s41598-019-45001-9. 10.1038/s41598-019-45001-9 31209263 PMC6572832

[11] Hutchison R.D. Steyn P.S. van Rensburg S.J.. Viridicatumtoxin, a new mycotoxin from *Penicillium viridicatum* Westling. Toxicol. Appl. Pharmacol. 24 (1973) 507–509. https://doi.org/10.1016/0041-008x(73)90057-4. 10.1016/0041-008x(73)90057-4 4122267

[12] Zheng C.-J. Yu H.-E. Kim E.-H. Kim W.-G.. Viridicatumtoxin B, a new anti-MRSA agent from *Penicillium* sp. FR11. J. Antibiot. 61 (2008) 633–637. https://doi.org/10.1038/ja.2008.84. 10.1038/ja.2008.84 19168978

[13] Nicolaou K.C. Nilewski C. Hale C.R.H. Ioannidou H.A. El Marrouni A. Koch L.G.. Total synthesis and structural revision of viridicatumtoxin B. Angew. Chem. Int. Ed. 52 (2013) 8736-8741. https://doi.org/10.1002/anie.201304691. 10.1002/anie.201304691 PMC3835450 23893651

[14] Nicolaou K.C. Hale C.R.H. Nilewski C. Ioannidou H.A. El Marrouni A. Nilewski L.G. Beabout K. Wang T.T. Shamoo Y.. Total synthesis of viridicatumtoxin B and analogues thereof: Strategy evolution, structural revision, and biological evaluation. J. Am. Chem. Soc. 136 (2014) 12137-12160. https://doi.org/10.1021/ja506472u. 10.1021/ja506472u 25317739 PMC4210137

[15] Drott M.T. Bastos R.W. Rokas A. Ries L.N.A. Gabaldón T. Goldman G.H. Keller N.P. Greco C.. Diversity of secondary metabolism in *Aspergillus nidulans* clinical isolates. mSphere 5 (2020) e00156-00120. https://doi.org/10.1128/mSphere.00156-20. 10.1128/mSphere.00156-20 32269157 PMC7142299

[16] Shang Z. Salim A.A. Khalil Z. Bernhardt P.V. Capon R.J.. Fungal biotransformation of tetracycline antibiotics. J. Org. Chem. 81 (2016) 6186–6194. https://doi.org/10.1021/acs.joc.6b01272. 10.1021/acs.joc.6b01272 27419475

[17] Li W. L. L. Zhang C. Cai Y. Gao Q. Wang F. Cao Y. JLin J. Li J. Shang Z. Lin W.. Investigations into the antibacterial mechanism of action of viridicatumtoxins. ACS Infect. Dis. 6 (2020) 1759–1769. https://doi.org/10.1021/acsinfecdis.0c00031. 10.1021/acsinfecdis.0c00031 32437130 PMC9040061

[18] Liu Y.-Y. Wang Y. Walsh T.R. Yi L.-X. Zhang R. Spencer J. Doi Y. Tian G. Dong B. Huang X. Yu L.-F. Gu D. Ren H. Chen X. Lv L. He D. Zhou H. Liang Z. Liu J.-H Shen J.. Emergence of plasmid-mediated colistin resistance mechanism MCR-1 in animals and human beings in China: a microbiological and molecular biological study. Lancet Infect. Dis. 16 (2016) 161-168. https://doi.org/10.1016/S1473-3099(15)00424-7. 10.1016/S1473-3099(15)00424-7 26603172

[19] Cherak Z. Loucif L. Moussi A. Rolain J.-M.. Epidemiology of mobile colistin resistance (mcr ) genes in aquatic environments. J. Global Antimicro. Resist. 27 (2021) 51-62. https://doi.org/10.1016/j.jgar.2021.07.021. 10.1016/j.jgar.2021.07.021 34438108

[20] Jiang X. Patil N.A. Azad M.A.K. Wickremasinghe H. Yu H. Zhao J. Zhang X. Li M. Gong B. Wan L. Ma W. Thompson P.E. Yang K. Yuan B. Schreiber F. Wang L. Velkov T. Roberts K.D. Li J. A novel chemical biology and computational approach to expedite the discovery of new generation polymyxins against life-threatening *Acinetobacter baumannii*. Chem. Sci. 12 (2021) 12211. https://doi.org/10.1039/d1sc03460j. 10.1039/d1sc03460j 34667587 PMC8457388

[21] Vaara M.. Polymyxins and their potential next generation as therapeutic antibiotics. Front. Microbiol. 10 (2019) 1689. https://doi.org/10.3389/fmicb.2019.01689. 10.3389/fmicb.2019.01689 31404242 PMC6671869

[22] Wang Z. Koirala B. Hernandez Y. Zimmerman M. Park S. Perlin D.S. Brady S.F.. A naturally inspired antibiotic to target multidrug-resistant pathogens. Nature 601 (2022) 606-611. https://doi.org/10.1038/s41586-021-04264-x. 10.1038/s41586-021-04264-x 34987225 PMC10321319

[23] Shlaes D.M. Shlaes J.H. Davies J. Williamson R.. Escherichia coli susceptible to glycopeptide antibiotics. Antimicro. Ag. Chemother. 33 (1989) 192-197. https://doi.org/10.1128/AAC.33.2.192. 10.1128/AAC.33.2.192 PMC171455 2655529

[24] Gordon N.C. Png K. Wareham D.W.. Potent synergy and sustained bactericidal activity of a vancomycin-colistin combination versus multidrug-resistant strains of *Acinetobacter baumannii*. Antimicro. Ag. Chemother. 54 (2010) 5316-5322. https://doi.org/10.1128/AAC.00922-10. 10.1128/AAC.00922-10 PMC2981237 20876375

[25] van Groesen E. Slingerland C.J. Innocenti P. Mihajlovic M. Masereeuw R. Martin N.I.. Vancomyxins: Vancomycin-polymyxin nonapeptide conjugates that retain anti-Gram-positive activity with enhanced potency against Gram-negative strains. ACS Infect. Dis. 7 (2021) 2746-2754. https://doi.org/10.1021/acsinfecdis.1c00318. 10.1021/acsinfecdis.1c00318 34387988 PMC8438664

[26] Hotchkiss R.D. Dubos R.J.. Fractionation of the bacteriocidal agent from cultures of a soil bacillus. J. Biol. Chem. 132 (1940) 791-792.

[27] Battersby A.R. Craig L.C.. The chemistry of tyrocidine. I. Isolation and characterization of a single peptide. J. Chem. Soc. 74 (1952) 4019-4023. https://doi.org/10.1021/ja01136a014. 10.1021/ja01136a014

[28] Gause G.F.. Gramicidin S and its use in the treatment of infected wounds. Nature 154 (1944) 703. https://doi.org/10.1038/154703a0. 10.1038/154703a0

[29] Gause G.F. Brazhnikova M.G.. Gramicidn S origin and mode of action. Lancet 244 (1944) 715-716. https://doi.org/10.1016/S0140-6736(00)88377-4. 10.1016/S0140-6736(00)88377-4

[30] Belozersky A.N. Passhina T.S.. Chemistry of gramacidin S. Lancet 244 (1944) 716-717. https://doi.org/10.1016/S0140-6736(00)88378-6. 10.1016/S0140-6736(00)88378-6

[31] Sergiev P.G.. Clinical use of gramicidin S. Lancet 244 (1944) 717-718. https://doi.org/10.1016/S0140-6736(00)88379-8. 10.1016/S0140-6736(00)88379-8

[32] Gause G.F.. Gramacidin S review of recent work. Lancet 248 (1946) 46-47. https://doi.org/10.1016/S0140-6736(46)90004-9. 10.1016/S0140-6736(46)90004-9

[33] Lazzaro B.P. Zasloff M. Rolff J.. Antimicrobial peptides: Application informed by evolution. Science 368 (2020) eaau5480. https://doi.org/10.1126/science.aau5480. 10.1126/science.aau5480 32355003 PMC8097767

[34] Zerweck J. Strandberg E. Kukharenko O. R. J. Bürck J. Wadhwani P. Ulrich A.S.. Molecular mechanism of synergy between the antimicrobial peptides PGLa and magainin 2. Sci. Rep. 7 (2017) 13153. https://doi.org/10.1038/s41598-017-12599-7. 10.1038/s41598-017-12599-7 29030606 PMC5640672

[35] Baharin N.H.Z. Mokhtar N.F.K. Nasir M. Desa M. Gopalsamy B. Zaki N.N.M. Yuswan M.H. Muthanna A.R. Dzaraly N.D. Abbasiliasi S. Hashim A.M. Sani M.S.A. Mustafa S.. The characteristics and roles of antimicrobial peptides as potential treatment for antibiotic-resistant pathogens: a review. PeerJ 9 (2021) e12193. https://doi.org/10.7717/peerj.12193. 10.7717/peerj.12193 35003909 PMC8679955

[36] Vanzolini T. Bruschi M. Rinaldi A.C. Magnani M. Fraternale A.. Multitalented synthetic antimicrobial peptides and their antibacterial, antifungal and antiviral mechanisms. Int. J. Mol. Sci. 23 (2022) 545. https://doi.org/10.3390/ijms23010545. 10.3390/ijms23010545 35008974 PMC8745555

[37] Klobucar K. Brown E.D.. New potentiators of ineffective antibiotics: Targeting the Gram-negative outer membrane to overcome intrinsic resistance. Curr. Opin. Chem. Biol. 66 (2022) 102099. https://doi.org/10.1016/j.cbpa.2021.102099. 10.1016/j.cbpa.2021.102099 34808425

[38] Gao B. Chen S. Hou Y.N. Zhao Y.J. Ye T. Xu Z.. Solution-phase total synthesis of teixobactin. Org. Biomol. Chem. 17 (2019) 1141-1153. https://doi.org/10.1039/c8ob02803f. 10.1039/c8ob02803f 30638238

[39] Ng V. Kuehne S.A. Chan W.C.. Rational design and synthesis of modified teixobactin analogues: *In vitro* antibacterial activity against *Staphylococcus aureus, Propionibacterium acnes* and *Pseudomonas aeruginosa*. Chem. Eur. J. 24 (2018) 9136-9147. https://doi.org/10.1002/chem.201801423. 10.1002/chem.201801423 29741277

[40] Charlop-Powers Z. Milshteyn A. Brady S.F.. Metagenomic small molecule discovery methods. Curr. Opin. Microbiol. 19 (2014) 70-75. https://doi.org/10.1016/j.mib.2014.05.021. 10.1016/j.mib.2014.05.021 25000402 PMC4135586

[41] Charlop-Powers Z. Brady S.F.. phylogeo: an R package for geographic analysis and visualization of microbiome data. Bioinformatics 31 (2015) 2909–2911. https://doi.org/10.1093/bioinformatics/btv269. 10.1093/bioinformatics/btv269 25913208 PMC4547612

[42] Charlop-Powers Z. Pregitzer C.C. Lemetre C. Ternei M.A. Maniko J. Hover B.M. Calle P.Y. McGuire K.L. Garbarino J. Forgione H.M. Charlop-Powers S. Brady S.F.. Urban park soil microbiomes are a rich reservoir of natural product biosynthetic diversity. Proc. Natl. Acad. Sci., U.S.A. 113 (2016) 14811-14816. https://doi.org/10.1073/pnas.1615581113. 10.1073/pnas.1615581113 27911822 PMC5187742

[43] Vila-Farres X. Chu J. Ternei M.A. Lemetre C. Park S. Perlin D.S. Brady S.F.. An optimized synthetic-bioinformatic natural product antibiotic sterilizes multidrug-resistant *Acinetobacter baumannii*-infected wounds. mSphere 3 (2018) 3:e00528-00517. https://doi.org/10.1128/mSphere.00528-17. 10.1128/mSphere.00528-17 PMC5784245 29404414

[44] Chu J. Koirala B. Forelli N. Vila-Farres X. Ternei M.A. Ali T. Colosimo D.A. Brady S.F.. Synthetic-bioinformatic natural product antibiotics with diverse modes of action. J. Am. Chem. Soc. 142 (2020) 14158-14168. https://doi.org/10.1021/jacs.0c04376. 10.1021/jacs.0c04376 32697091 PMC8011376

[45] Hover B.M. Kim S.-H. Katz M. Charlop-Powers Z. Owen J.G. Ternei M.A. Maniko J. Estrela A. B. Molina H. Park S. Perlin D.S. Brady S.F.. Culture-independent discovery of the malacidins as calcium-dependent antibiotics with activity against multidrug-resistant Gram-positive pathogens. Nat. Microbiol. 3 (2018) 415-422. https://doi.org/10.1038/s41564-018-0110-1. 10.1038/s41564-018-0110-1 29434326 PMC5874163

[46] Sun Z. Shang Z. Forelli N. Po K.H.L. Chen S. Brady S.F. Li X.. Total synthesis of malacidin A by beta-hydroxyaspartic acid ligation-mediated cyclization and absolute structure establishment. Angew. Chem. Int. Ed. 59 (2020) 19868–19872. https://doi.org/10.1002/anie.202009092. 10.1002/anie.202009092 PMC8130013 32725837

[47] Kovalenko N. Howard G.K. Swain J.A. Hermant Y. Cameron A.J. Cook G.M. Ferguson S.A. Stubbing L.A. Harris P.W.A. Brimble M.A.. A concise synthetic strategy towards the novel calcium-dependent lipopeptide antibiotic, malacidin A and analogues. Front. Chem. 9 (2021) 687875. https://doi.org/10.3389/fchem.2021.687875. 10.3389/fchem.2021.687875 34422759 PMC8372822

[48] Upert G. Luther A. Obrecht D. Ermert P.. Emerging peptide antibiotics with therapeutic potential. Medicine Drug Discov. 9 (2021) 100078. https://doi.org/10.1016/j.medidd.2020.100078. 10.1016/j.medidd.2020.100078 PMC7773004 33398258

[49] Liu Y. Shi J. Tong Z. Jia Y. Yang B. Wang Z.. The revitalization of antimicrobial peptides in the resistance era. Pharmacol. Res. 163 (2021) 105276. https://doi.org/10.1016/j.phrs.2020.105276. 10.1016/j.phrs.2020.105276 33161137

[50] Zhu Y. Hao W. Wang X. Ouyang J. Deng X. Yu H. Wang Y.. Antimicrobial peptides, conventional antibiotics, and their synergistic utility for the treatment of drug-resistant infections. Med. Res. Rev. (2022). https://doi.org/10.1002/med.21879. 10.1002/med.21879 34984699

[51] Nicolaou K.C. Rigol S.. Perspectives from nearly five decades of total synthesis of natural products and their analogues for biology and medicine. Nat. Prod. Rep. 37 (2020) 1404-1435. https://doi.org/10.1039/d0np00003e. 10.1039/d0np00003e 32319494 PMC7578074

[52] Wender P.A. Hardman C.T. Ho S. Jeffreys M.S. Maclaren J.K. Quiroz R.V. Ryckbosch S.M. Shimizu A.J. Sloane J.L. Stevens M.C.. Scalable synthesis of bryostatin 1 and analogs, adjuvant leads against latent HIV. Science 358 (2017) 218-223. https://doi.org/10.1126/science.aan7969. 10.1126/science.aan7969 29026042 PMC5714505

